# Supplementary material for: The causal relationship between the human gut microbiota and pyogenic arthritis: a Mendelian randomization study
Source: Front Cell Infect Microbiol. 2024 Nov 26;14:1452480. doi: 10.3389/fcimb.2024.1452480 (PMC11629706; doi:10.3389/fcimb.2024.1452480)
Supplement: Supplementary file 1 [file DataSheet1.zip › mendelian test/ebi-a-GCST90027775.csv_forest.pdf]

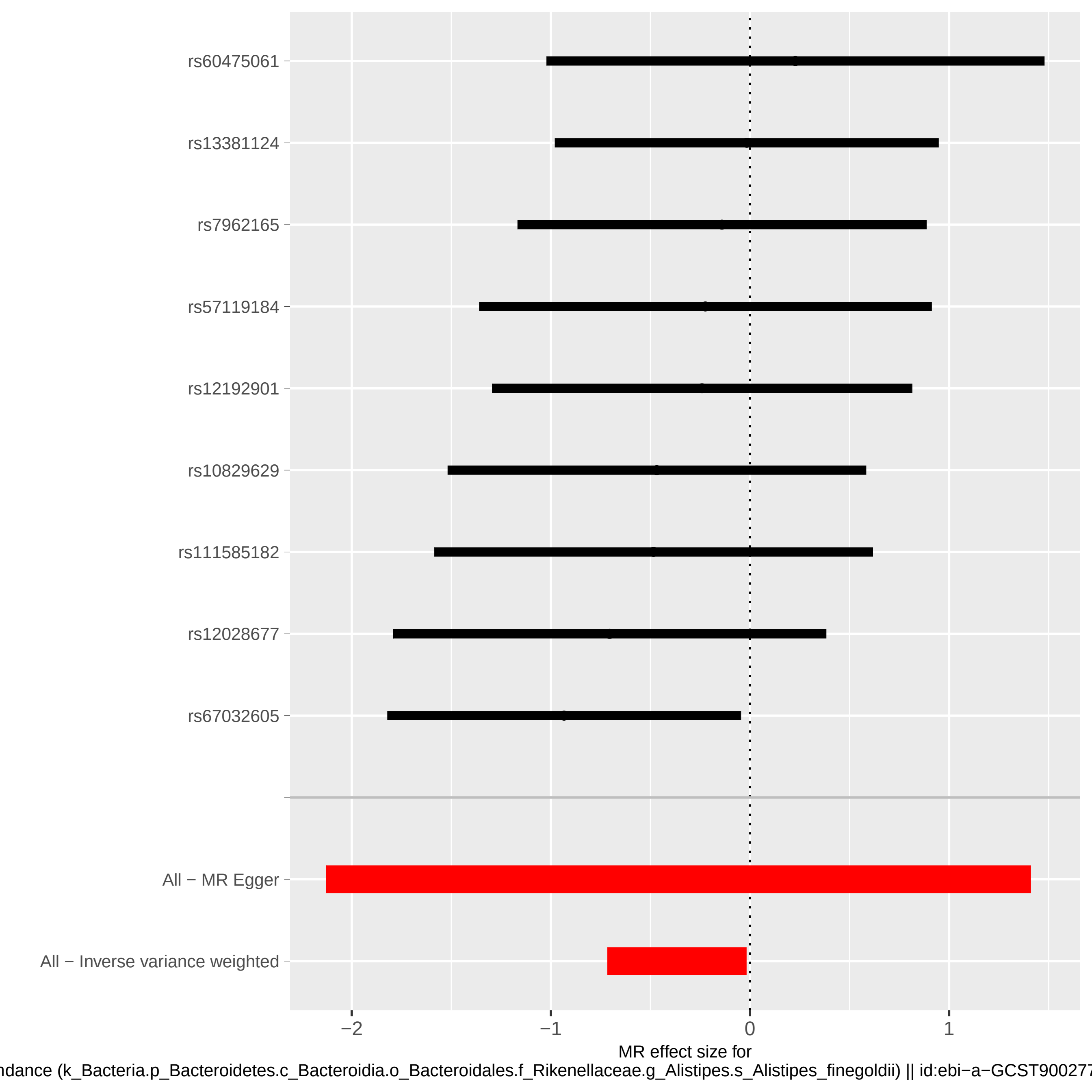

rs60475061
rs13381124
rs7962165
rs57119184
rs12192901
rs10829629
rs111585182
rs12028677
rs67032605
All − MR Egger
All − Inverse variance weighted
−2
−1
0
1
MR effect size for
ndance (k_Bacteria.p_Bacteroidetes.c_Bacteroidia.o_Bacteroidales.f_Rikenellaceae.g_Alistipes.s_Alistipes_finegoldii) || id:ebi−a−GCST90027
